# Factors that affect scientific publication in Africa—A gender perspective

**DOI:** 10.3389/frma.2023.1040823

**Published:** 2023-02-20

**Authors:** Catherine Beaudry, Heidi Prozesky, Carl St-Pierre, Seyed Reza Mirnezami

**Affiliations:** ^1^Department of Mathematics and Industrial Engineering, Polytechnique Montreal, Montreal, QC, Canada; ^2^Centre Interuniversitaire de Recherche sur la Science et la Technologie (CIRST), Université du Québec à Montréal, Montreal, QC, Canada; ^3^Centre for Research on Evaluation, Science and Technology (CREST) and DSI-NRF Centre of Excellence in Scientometrics and STI Policy, Stellenbosch University, Stellenbosch, South Africa; ^4^Research Institute for Science, Technology, and Industrial Policy (RISTIP), Sharif University of Technology, Tehran, Iran

**Keywords:** Africa, gender difference, scientific publication output, workload, funding, career stage, collaboration, mobility

## Abstract

A large body of literature on gender differences in scientific publication output has clearly established that women scientists publish less that men do. Yet, no single explanation or group of explanations satisfactorily accounts for this difference, which has been called the “productivity puzzle”. To provide a more refined portrait of the scientific publication output of women in relation to that of their male peers, we conducted a web-based survey in 2016 of individual researchers across all African countries, except Libya. The resulting 6,875 valid questionnaires submitted by respondents in the STEM, Health Science and SSH fields were analyzed using multivariate regressions on the self-reported number of articles published in the preceding 3 years. Controlling for a variety of variables including career stage, workload, mobility, research field, and collaboration, we measured the direct and moderating effect of gender on scientific production of African researchers. Our results show that, while women's scientific publication output is positively affected by collaboration and age (impediments to women's scientific output decrease later in their careers), it is negatively impacted by care-work and household chores, limited mobility, and teaching hours. Women are as prolific when they devote the same hours to other academic tasks and raise the same amount of research funding as their male colleagues. Our results lead us to argue that the standard academic career model, relying on continuous publications and regular promotions, assumes a masculine life cycle that reinforces the general perception that women with discontinuous careers are less productive than their male colleagues, and systematically disadvantages women. We conclude that the solution resides beyond women's empowerment, i.e., in the broader institutions of education and the family, which have an important role to play in fostering men's equal contribution to household chores and care-work.

## Introduction

Scientific publication output remains a yardstick for academic promotion, even in academic contexts that do not appear to support research, as is often the case in Africa (Teferra and Altbachl, 2004). African research currently accounts for a fraction (<3%) of global scientific publications (Soete et al., 2015), much smaller than is desirable if Africa's researchers' potential contribution is to be realized for the benefit of its populations (Adams et al., 2010). At the same time, one of the most serious gaps that African universities need to close if African countries are to utilize the full potential of their human capital is the gender gap in research participation. Globally, women account for a minority (28.8%) of the world's researchers (Unesco, 2017) and approximately 24.0% of researchers in African countries (Npca, 2014).

More importantly, and the focus of this paper, the consistent finding globally is that the scientific publication output of this minority of female researchers is lower than that of men (Larivière et al., 2013; Huyer, 2015), even when counting both single-author and mixed-gender multiple-author papers, but the gap is narrowing. For instance, Önder and Yilmazkuday (2020) showed that since the early 2000s, there is no significant difference between the number of papers published by all-women and mixed-gender teams compared to all-men teams. In addition, women are now more prevalent than in the past as single authors and as first authors of mixed-author papers (Holman et al., 2018). However, research on scientific publication output of researchers in Africa, especially in gender-disaggregated form, is scarce, partly because, until recently, women scientists were “so rare in Africa as to be nearly invisible” (Campion and Shrum, 2004, p. 460). A review of the literature shows that even after half a century of empirical research on gender differences in scientific publication output mostly conducted in developed countries (Prozesky, 2006), no single explanation or group of explanations satisfactorily accounts for the phenomenon, aptly referred to by Cole and Zuckerman (1984) as the “productivity puzzle”.

This study is based on arguably the largest survey ever conducted amongst African scientists, and specifically young African scientists. The survey goal was to provide evidence and arguments for improving current institutional policies in African countries. Respondents were asked to provide information about their publications, funding, collaboration, mobility, and family situation. In this article, we compare the scientific publication output of men and women while controlling for a variety of variables including career stage, workload, mobility, research field, and collaboration. As such, we provide a more refined portrait of the scientific publication output of women compared to that of their male peers. As such, we respond to what has been referred to as “an urgent need for more gender-disaggregated data and more refined statistical analysis” to facilitate the formulation and implementation of effective institutional change strategies toward gender equality in research participation and scientific publication output, specifically in the African context (Mama, 2003, p. 120).

## Theoretical framework

A large body of literature on gender differences in scientific publication output, recently reviewed in a global meta-analysis of 110 published studies by Astegiano et al. (2019), has clearly established that women scientists publish less that men do. In fact, the lesser scientific publication output of women scientists is an important topic in the science-policy academic community. Several high-quality studies have investigated the gender difference, providing evidence from different fields and geographical areas. Boekhout et al. (2021) found that women are less likely to continue their career as publishing researchers than men, with an average difference of 15% between their publication output. The gap is even more visible in Engineering. A study by Ghiasi et al. (2015) showed that men dominate 80% of all the scientific production in Engineering, where there is a reproduction of the male-dominated scientific structures through repeating of predominantly male-driven collaborations. Some studies in Health Science, however, suggest that the increase in first and last authorship by women implies an improvement in gender advancement (Boekhout et al., 2021; Malchuk et al., 2021).

Beyond its description, the literature provides interesting justifications for this gender gap or disparity. Traag and Waltman (2022) propose the broader concept of gender disparity, i.e., a causal effect that also includes a bias, which they suggest defining “as a direct causal effect that is unjustified”. Derrick et al. (2021) used a global survey of 11,226 academic parents to investigate the effect of parental responsibilities and parental leave on research output and academic productivity. Their results suggest that parental engagement explains gender differences in academic productivity, which is compatible with findings by Morgan et al. (2021). From a different perspective, Huang et al. (2020) offered a longitudinal picture of gender differences in research performance through a bibliometric analysis of over 1.5 million gender-identified authors. They found that men and women publish at a comparable annual rate but the gender gap originates from differences in publishing, career lengths and dropout rates.

However, there is still no consensus on the reasons for gender differences in research output (Larivière et al., 2013). A number of conceptual frameworks have summarized attempts at explaining the productivity puzzle (e.g., Cole and Zuckerman, 1984; Ceci et al., 2014; Van Den Besselaar and Sandström, 2017). The most recent global framework (Halevi, 2019) groups the explanations as follows: (1) underrepresentation (of women in many areas of science); (2) career development (women's careers are impacted more by marriage and starting a family); (3) specialization vs. diversification (women prefer the latter to the former); (4) collaboration and professional networks (women prefer domestic, smaller and homogenous networks); and (5) research vs. teaching (women focus on teaching and service rather than research).

Most of the research on the gender gap in research output in Africa has thus far focused on South Africa. Rossello (2021) observed a lower publication output of female PhD students mostly when they are supervised by men, which could be due to female PhD students' early motherhood being accommodated differently by male and female supervisors. Differential family commitments at other career stages have also been linked to women's lower research output (Cilliers et al., 2008; Bhana and Pillay, 2012; Boshoff and Bosch, 2012; Mokone et al., 2012; Mohope, 2014; Ramnund-Mansingh and Seedat-Khan, 2020). Obers (2014) and Obers (2015) found that family responsibilities prevent travel to conferences, which limited access to supportive disciplinary networks (the accumulation of social capital), and in turn, research output. Family responsibilities also make it difficult for women to have uninterrupted periods of time that they can dedicate to research. The role of Science Granting Councils^1^ (SGCs), as providers of researcher grants, scholarships and funding for science in general (see Mouton, 2019, for instance), in propagating the gender gap is also mentioned in the literature. Jackson et al. (2022) assessed the sub-Saharan Africa's SGCs and the status of gender research and collaboration. Their results indicate that most of the SGCs had very limited or no gender-related funding programs to deal with the challenges that women scholars face.

Further research published on South African female academics suggests, as explanations for the gender gap in research output, their preference for teaching over research (even viewing teaching as a “calling”), together with a lack of confidence, skills, capacity, and commitment to undertake and publish research (Schulze, 2005; Boshoff and Bosch, 2012; Garnett and Mahomed, 2012; Obers, 2014, 2015; Callaghan, 2017; Chitsamatanga and Rembe, 2019; Ramohai, 2019). In addition to either voluntarily-chosen or allocated teaching commitments, heavy administrative workloads have also been mentioned (Zulu, 2013; Obers, 2015) or identified as the main barrier to publishing among women (Garnett and Mahomed, 2012).

A study among women scientists in other countries in Africa, namely Ghana and Kenya, as well as India, found, contrary to the global pattern, that men and women display similar levels of self-reported scientific publication output (Campion and Shrum, 2004). In the case of Ghana, faculty members claim that career interruptions due to childbirth overlap with the “golden periods” for establishing a good track-record of research output, which is not the case for their male colleagues (Ayentimi and Abadi, 2022). A recent study by Sougou et al. (2022) on Ghana, Senegal, Burkina Faso, Niger and Mali identifies four themes associated with barriers to women's careers development: (1) family- and community-related barriers; (2) organizational culture and institutional policies that maintain gender inequalities and a glass ceiling for women; (3) the lack for women-appropriate empowerment programs in research, rather than the spouse-relationship management programs that seem the norm; and (4) the classic impostor syndrome that women suffer from.

According to Zulu (2013, p. 758), nowhere are culturally defined gender roles and gender socialization patterns that associate women with home-making “more sharply crystallized than in systems organized along patriarchal lines such as those of South Africa and other African countries”. But in South Africa, as elsewhere, tensions between women's caretaking of children and delivering on research output have been heightened during the COVID-19 pandemic. In a study by Walters et al. (2021), several women referred explicitly to the uninterrupted research productivity of men over the lockdown period, relating this to traditional gender-role expectations of their cultures.

It has also been suggested that specifically Black African women academics in South Africa may feel morally pressured to engage in academic “care work”, especially toward Black working-class students. Such a carer role is stereotypically associated with their social and cultural backgrounds, but inadvertently deadlocks them in “the bottom of the knowledge production hierarchy” (Magoqwana et al., 2019, p. 6). However, the gender gap exists “even among elite scientists”, such as holders of South Africa Research Chairs, and cannot be attributed to differences in the lengths of scientists' careers (Sá et al., 2020, p. 1). In fact, Boshoff and Bosch (2012) posit that women tend to lose research momentum when they re-enter the research track after childbearing.

In South Africa, women's younger career age, lower qualifications, lower rank or contract status, and lack of experience have further been cited as causes of the gender gap in research output (Wolhuter et al., 2013; Zulu, 2013; Callaghan, 2017). Intersectionality with class and race is also important in this regard: working-class Black women academics are “latecomers” in the academy and therefore mostly junior in academic positions. Their teaching workloads tend to be much heavier than those of their “privileged colleagues who have ‘paid their dues' and are thus exempt from heavy teaching burdens”, leading to many of them struggling to participate in academic research (Magoqwana et al., 2019, p. 16). As Waltman (2021) justly argues, the topic of gender disparities in science is “highly complex, and each individual study can provide insight into only a small part of a very complicated puzzle”. We do not claim to have all the answers, but we have accounted for as many as the factors that we could include in our analysis. As such, this study sheds some lights on interactive effects of gender and other determinants of scientific publication.

## Data and methodology

Data for the study were collected using a web-based, self-administered questionnaire, a previous version of which had been pre-tested for the purpose of a 2013 worldwide survey (Friesenhahn and Beaudry, 2014). Following this pre-test, corrections were made, and the questionnaire was re-tested in Indonesia, Malaysia, Singapore and Thailand in a further study in 2015. It was then shortened and adapted to the African context and tested in Zambia in early 2016. Later in 2016, we administered a bilingual (French and English) version of the questionnaire to more than 120,000 email addresses of individuals across all African countries^2^ that had co-authored at least one scientific article in a journal indexed by Clarivate™ Web of Science in the preceding 10 years^3^. In total, 7,515 individuals responded, constituting a response rate of approximately 10%, once multiple email addresses of some individuals were excluded. Removing individuals not working in Africa, and those for which there are missing values on some of the explanatory variables (for instance, more than 1,000 individuals did not provide their age, or their gender)^4^, our final sample contains 4,676 observations of which 2,562 are in Science, Technology, Engineering and Mathematics—STEM—and 1,495 are Health Scientists, and 619 are in the Social Science and Humanities (SSH)^5^. Table 3 in the Appendix lists the number of respondents per country in that final sample used for the regression analysis.

We used ordinary least square regression analysis to identify the main determinants of scientific output in Africa. The dependent variable of our model is the self-reported number of scientific articles published (or accepted for publication) in the 3 years preceding the survey (i.e., 2013–2015), which we transformed using the natural logarithm to obtain a variable that follows a normal distribution^6^. The continuous variables on the right-hand side of the regression equation include the amount of funding in dollars and the time spent on different academic tasks measured by the number of hours per week spent on different academic tasks (i.e., teaching, supervising, research, admin, service, consultation, and fundraising). These continuous variables were similarly transformed using the natural logarithm. Other right-hand side variables are dummy variables identifying (1) whether the researcher has the main care responsibility; (2) the career stage of the researcher (early, mid, or late)^7^; (3) whether the researcher is/was mobile during study/work; (4) whether the researcher has any collaboration with other scientists in their own institution, in their own country, in Africa, or outside of Africa; and (5) research field. Table 4 in the Appendix describes the variables used in the study.

## Descriptive statistics

Our sample is composed of 1,436 women (30.7%) women and 3,240 men (69.3%). The average age of the researchers surveyed is 46.2, women being slightly younger (but the difference is not significant). Although these researchers on average contribute to 42.8% of housework and care-work, women report contributing to 68.0% of these chores, compared to men's 31.6%. On average, women published 7.1 articles over the 3 years preceding the survey, compared to 8.9 articles by men. On average, these individuals had access to approximately 82,500$ of research funds over the same period, with both women and men raising similar amounts—the gender difference is not statistically significant, but the average for women is slightly higher than for men, as also shown by Prozesky and Mouton (2019).

In addition, 26.9% of the women and 40.1% of the men surveyed had studied in another country for their highest qualification, and 26.6% of women and 36.5% of men had worked abroad in the 3 years preceding the survey. These researchers often collaborated with colleagues from their own institutions (61.0%), sometimes with colleagues in their own country (35.2%) and internationally (37.5%), but rarely with those in other African countries (15.2%). Furthermore, men systematically declared more frequent collaboration than women.

The task that occupied the survey respondents most was undoubtedly research (on average 10.6 h a week), followed by teaching (7.9 h a week), administration (5.7 h a week) and supervising graduate students (5.7 h a week). The number of working hours devoted to various academic tasks is where we also note significant gender differences. On average, women spend less time on teaching and consulting than men, but more time on all the other tasks than their male counterparts do. From another perspective, we may put activities in three broad categories: (1) research, supervision, and fundraising (knowledge advancement); (2) teaching and consultation (knowledge dissemination); and (3) administration and service (building scientific community). Classified as such, women spend 19.2 h per week on knowledge advancement (1.6 h per week more than men). On knowledge dissemination, women and men spend 8.9 h and 10.1 h per week, respectively. On the third category of academic tasks, building scientific community, women spend more time (9.6 h per week) than men do (7.8 h per week).

Thus, if it were only a matter of available time for research, women would publish more, but other confounding factors need to be taken into consideration at the same time. Some of these are the disciplines^8^ in which these individuals work: men are concentrated in the STEM fields (57.6% in STEM compared to 21.1% in Health Sciences and 21.3% in SSH), whereas women are spread somewhat more evenly across the three fields (44.4% in STEM, 25.8% in Health and 29.7% in SSH). The next section presents our regression results where all these factors are explored collectively.

## Results

The descriptive statistics presented in the previous section clearly highlight important gender differences both in terms of the number of publications both in the factors that may contribute to this gap. As most of these factors are likely to collectively influence scientific publication output, this section discusses the results obtained using multivariate regression analysis (ordinary least squares) on the natural logarithm of the number of articles. The results are presented in Tables 1 and 2.

**Table 1:** Regression results.

**Variables**	**(1)**	**(2)**	**(3)**	**(4)**	**(5)**	**(6)**	**(7)**	**(8)**	**(9)**
Main care & housework	−0.0353^**^	−0.0341^**^	−0.0347^**^	−0.0357^**^	−0.0350^**^	−0.0332^**^	−0.0351^**^	−0.0349^**^	−0.0329^*^
	(0.0168)	(0.0168)	(0.0168)	(0.0168)	(0.0168)	(0.0168)	(0.0168)	(0.0168)	(0.0168)
Early–career researcher^a^	−0.1483^***^	−0.1492^***^	−0.1488^***^	−0.1486^***^	−0.1490^***^	−0.1481^***^	−0.1482^***^	−0.1466^***^	−0.1486^***^
	(0.0204)	(0.0204)	(0.0204)	(0.0204)	(0.0204)	(0.0204)	(0.0204)	(0.0204)	(0.0204)
Mid–career researcher^a^	−0.0158	−0.0172	−0.0162	−0.0157	−0.0160	−0.0154	−0.0157	−0.0142	−0.0139
	(0.0193)	(0.0193)	(0.0193)	(0.0193)	(0.0193)	(0.0193)	(0.0193)	(0.0193)	(0.0193)
Teaching hours^b^	0.0144	0.0138	0.0326^**^	0.0145	0.0150	0.0137	0.0144	0.0142	0.0141
	(0.0115)	(0.0115)	(0.0134)	(0.0115)	(0.0115)	(0.0115)	(0.0115)	(0.0115)	(0.0115)
Supervising hours^b^	0.2185^***^	0.2184^***^	0.2191^***^	0.2006^***^	0.2190^***^	0.2182^***^	0.2186^***^	0.2192^***^	0.2195^***^
	(0.0146)	(0.0146)	(0.0145)	(0.0171)	(0.0146)	(0.0145)	(0.0146)	(0.0146)	(0.0145)
Research hours^b^	0.0230^*^	0.0217^*^	0.0215^*^	0.0236^*^	0.0042	0.0232^*^	0.0231^*^	0.0237^*^	0.0225^*^
	(0.0125)	(0.0125)	(0.0125)	(0.0125)	(0.0148)	(0.0125)	(0.0125)	(0.0125)	(0.0125)
Admin hours^b^	−0.0427^***^	−0.0431^***^	−0.0420^***^	−0.0435^***^	−0.0427^***^	−0.0608^***^	−0.0428^***^	−0.0420^***^	−0.0432^***^
	(0.0124)	(0.0124)	(0.0124)	(0.0124)	(0.0124)	(0.0145)	(0.0124)	(0.0124)	(0.0124)
Service hours^b^	0.0316^*^	0.0317^*^	0.0309^*^	0.0324^*^	0.0323^*^	0.0302^*^	0.0263	0.0320^*^	0.0314^*^
	(0.0167)	(0.0167)	(0.0167)	(0.0167)	(0.0167)	(0.0167)	(0.0202)	(0.0167)	(0.0166)
Consultation hours^b^	−0.0592^***^	−0.0590^***^	−0.0588^***^	−0.0586^***^	−0.0579^***^	−0.0578^***^	−0.0589^***^	−0.0818^***^	−0.0583^***^
	(0.0165)	(0.0165)	(0.0165)	(0.0165)	(0.0165)	(0.0165)	(0.0165)	(0.0194)	(0.0165)
Fundraising hours^b^	0.0824^***^	0.0816^***^	0.0821^***^	0.0832^***^	0.0824^***^	0.0819^***^	0.0823^***^	0.0816^***^	0.0408^*^
	(0.0213)	(0.0213)	(0.0213)	(0.0213)	(0.0213)	(0.0213)	(0.0213)	(0.0213)	(0.0245)
Funding^b^	0.0152^***^	0.0129^***^	0.0153^***^	0.0151^***^	0.0150^***^	0.0152^***^	0.0152^***^	0.0152^***^	0.0151^***^
	(0.0019)	(0.0021)	(0.0019)	(0.0019)	(0.0019)	(0.0019)	(0.0019)	(0.0019)	(0.0019)
Study–related mobility	−0.0337^**^	−0.0331^*^	−0.0337^**^	−0.0331^*^	−0.0344^**^	−0.0343^**^	−0.0338^**^	−0.0333^*^	−0.0333^**^
	(0.0170)	(0.0170)	(0.0170)	(0.0170)	(0.0170)	(0.0170)	(0.0170)	(0.0170)	(0.0170)
Work–related mobility	0.0230	0.0236	0.0226	0.0233	0.0237	0.0228	0.0230	0.0228	0.0233
	(0.0174)	(0.0174)	(0.0174)	(0.0174)	(0.0174)	(0.0174)	(0.0174)	(0.0174)	(0.0174)
Collab. with own inst.	0.0913^***^	0.0912^***^	0.0910^***^	0.0909^***^	0.0915^***^	0.0914^***^	0.0915^***^	0.0920^***^	0.0910^***^
	(0.0169)	(0.0169)	(0.0169)	(0.0169)	(0.0169)	(0.0169)	(0.0169)	(0.0169)	(0.0169)
Collab. with own country	0.0881^***^	0.0880^***^	0.0871^***^	0.0888^***^	0.0882^***^	0.0883^***^	0.0881^***^	0.0880^***^	0.0888^***^
	(0.0177)	(0.0177)	(0.0177)	(0.0177)	(0.0177)	(0.0177)	(0.0177)	(0.0177)	(0.0176)
Collab. within Africa	0.0973^***^	0.0968^***^	0.0984^***^	0.0966^***^	0.0981^***^	0.0967^***^	0.0973^***^	0.0973^***^	0.0992^***^
	(0.0237)	(0.0237)	(0.0237)	(0.0237)	(0.0237)	(0.0237)	(0.0237)	(0.0237)	(0.0237)
Collab. outside Africa	0.0684^***^	0.0677^***^	0.0685^***^	0.0681^***^	0.0680^***^	0.0682^***^	0.0684^***^	0.0685^***^	0.0665^***^
	(0.0177)	(0.0176)	(0.0176)	(0.0176)	(0.0176)	(0.0176)	(0.0177)	(0.0176)	(0.0176)
NOT South Africa x STEM^c^	0.1994^***^	0.1990^***^	0.1949^***^	0.2014^***^	0.1993^***^	0.2030^***^	0.1994^***^	0.1995^***^	0.1977^***^
	(0.0277)	(0.0277)	(0.0278)	(0.0277)	(0.0277)	(0.0278)	(0.0277)	(0.0277)	(0.0277)
NOT South Africa x Health ^c^	0.3160^***^	0.3156^***^	0.3131^***^	0.3171^***^	0.3163^***^	0.3209^***^	0.3162^***^	0.3146^***^	0.3133^***^
	(0.0313)	(0.0313)	(0.0313)	(0.0313)	(0.0313)	(0.0313)	(0.0313)	(0.0313)	(0.0312)
NOT South Africa x SSH ^c^	0.1305^***^	0.1312^***^	0.1268^***^	0.1326^***^	0.1308^***^	0.1340^***^	0.1306^***^	0.1316^***^	0.1279^***^
	(0.0334)	(0.0334)	(0.0334)	(0.0334)	(0.0334)	(0.0334)	(0.0334)	(0.0334)	(0.0334)
South Africa x STEM ^c^	0.0761^**^	0.0766^**^	0.0728^**^	0.0790^***^	0.0762^**^	0.0786^***^	0.0763^**^	0.0776^**^	0.0748^**^
	(0.0304)	(0.0303)	(0.0304)	(0.0304)	(0.0303)	(0.0304)	(0.0304)	(0.0304)	(0.0303)
South Africa x Health ^c^	0.1509^***^	0.1499^***^	0.1457^***^	0.1515^***^	0.1495^***^	0.1528^***^	0.1506^***^	0.1504^***^	0.1476^***^
	(0.0376)	(0.0376)	(0.0377)	(0.0376)	(0.0376)	(0.0376)	(0.0376)	(0.0376)	(0.0376)
Woman	−0.1191^***^	−0.1709^***^	0.0029	−0.2233^***^	−0.2561^***^	−0.2335^***^	−0.1433^***^	−0.2272^***^	−0.3085^***^
	(0.0187)	(0.0297)	(0.0489)	(0.0552)	(0.0607)	(0.0512)	(0.0550)	(0.0525)	(0.0584)
Woman x Funding^b^		0.0078^**^							
		(0.0035)							
Woman x Teaching hours^b^			−0.0594^***^						
			(0.0220)						
Woman x Supervising hours^b^				0.0525^**^					
				(0.0262)					
Woman x Research hours^b^					0.0576^**^				
					(0.0243)				
Woman x Admin hours^b^						0.0585^**^			
						(0.0244)			
Woman x Service hours^b^							0.0152		
							(0.0326)		
Woman x Consultation hours^b^								0.0783^**^	
								(0.0356)		
Woman x Fundraising hours^b^									0.1301^***^	
									(0.0381)
Constant	1.4606^***^	1.4813^***^	1.4271^***^	1.4905^***^	1.5008^***^	1.4924^***^	1.4678^***^	1.4874^***^	1.5208^***^
	(0.0626)	(0.0632)	(0.0638)	(0.0643)	(0.0648)	(0.0639)	(0.0645)	(0.0637)	(0.0649)
Number of observations	4,676	4,676	4,676	4,676	4,676	4,676	4,676	4,676	4,676
F	55.4716	53.4156	53.5351	53.3622	53.4473	53.4551	53.1604	53.4063	53.7695
R^2^	0.2152	0.2161	0.2165	0.2159	0.2162	0.2162	0.2153	0.216	0.2172
Adjusted R^2^	0.2113	0.212	0.2124	0.2119	0.2121	0.2122	0.2112	0.212	0.2132
Log-likelihood	−3,656.63	−3,654.11	−3,652.98	−3,654.61	−3,653.81	−3,653.74	−3,656.52	−3,654.20	−3,650.76

***, **, * Represent significance at the 0.01, 0.05 and 0.1 levels; Standard errors in parentheses.

x Represents the interaction between two variables.

a Late-career researcher is the omitted career-stage variable.

b All continuous variables were transformed using the natural logarithm: ln (*variable* + 1).

c South Africa x SSH fields is the omitted country x disciplinary field interactive variable.

**Table 2:** Regression results (continued).

**Variables**	**(10)**	**(11)**	**(12)**	**(13)**	**(14)**	**(15)**	**(16)**	**(17)**
Main care & housework		−0.0352^**^	−0.0353^**^	−0.0358^**^	−0.0353^**^	−0.0354^**^	−0.0354^**^	−0.0356^**^
		(0.0168)	(0.0168)	(0.0168)	(0.0168)	(0.0168)	(0.0168)	(0.0168)
Early–career researcher^a^	−0.1487^***^		−0.1484^***^	−0.1479^***^	−0.1483^***^	−0.1479^***^	−0.1471^***^	−0.1485^***^
	(0.0204)		(0.0204)	(0.0204)	(0.0204)	(0.0204)	(0.0204)	(0.0204)
Mid–career researcher^a^	−0.0156		−0.0158	−0.0155	−0.0157	−0.0155	−0.0153	−0.0159
	(0.0193)		(0.0193)	(0.0193)	(0.0193)	(0.0193)	(0.0193)	(0.0193)
Teaching hours^b^	0.0143	0.0139	0.0143	0.0145	0.0142	0.0147	0.0143	0.0144
	(0.0115)	(0.0115)	(0.0115)	(0.0115)	(0.0115)	(0.0115)	(0.0115)	(0.0115)
Supervising hours^b^	0.2192^***^	0.2182^***^	0.2186^***^	0.2187^***^	0.2186^***^	0.2187^***^	0.2186^***^	0.2184^***^
	(0.0146)	(0.0146)	(0.0146)	(0.0146)	(0.0146)	(0.0146)	(0.0145)	(0.0146)
Research hours^b^	0.0233^*^	0.0231^*^	0.0228^*^	0.0230^*^	0.0229^*^	0.0229^*^	0.0230^*^	0.0227^*^
	(0.0125)	(0.0125)	(0.0125)	(0.0125)	(0.0125)	(0.0125)	(0.0125)	(0.0125)
Admin hours^b^	−0.0433^***^	−0.0432^***^	−0.0428^***^	−0.0428^***^	−0.0427^***^	−0.0425^***^	−0.0429^***^	−0.0427^***^
	(0.0124)	(0.0124)	(0.0124)	(0.0124)	(0.0124)	(0.0124)	(0.0124)	(0.0124)
Service hours^b^	0.0311^*^	0.0313^*^	0.0316^*^	0.0317^*^	0.0314^*^	0.0319^*^	0.0323^*^	0.0320^*^
	(0.0167)	(0.0167)	(0.0167)	(0.0167)	(0.0167)	(0.0167)	(0.0167)	(0.0167)
Consultation hours^b^	−0.0594^***^	−0.0603^***^	−0.0591^***^	−0.0592^***^	−0.0594^***^	−0.0591^***^	−0.0593^***^	−0.0590^***^
	(0.0165)	(0.0165)	(0.0165)	(0.0165)	(0.0165)	(0.0165)	(0.0165)	(0.0165)
Fundraising hours^b^	0.0810^***^	0.0818^***^	0.0823^***^	0.0819^***^	0.0825^***^	0.0825^***^	0.0825^***^	0.0816^***^
	(0.0213)	(0.0213)	(0.0213)	(0.0213)	(0.0213)	(0.0213)	(0.0213)	(0.0213)
Funding^b^	0.0151^***^	0.0153^***^	0.0152^***^	0.0152^***^	0.0152^***^	0.0151^***^	0.0151^***^	0.0152^***^
	(0.0019)	(0.0019)	(0.0019)	(0.0019)	(0.0019)	(0.0019)	(0.0019)	(0.0019)
Study–related mobility	−0.0339^**^	−0.0330^*^		−0.0335^**^	−0.0337^**^	−0.0337^**^	−0.0333^**^	−0.0334^**^
	(0.0170)	(0.0170)		(0.0170)	(0.0170)	(0.0170)	(0.0170)	(0.0170)
Work–related mobility	0.0237	0.0226	0.0231		0.0231	0.0229	0.0228	0.0233
	(0.0174)	(0.0174)	(0.0174)		(0.0174)	(0.0174)	(0.0174)	(0.0174)
Collab. with own inst.	0.0913^***^	0.0915^***^	0.0913^***^	0.0912^***^		0.0917^***^	0.0916^***^	0.0908^***^
	(0.0169)	(0.0169)	(0.0169)	(0.0169)		(0.0169)	(0.0169)	(0.0169)
Collab. with own country	0.0884^***^	0.0876^***^	0.0881^***^	0.0882^***^	0.0879^***^		0.0883^***^	0.0877^***^
	(0.0177)	(0.0177)	(0.0177)	(0.0177)	(0.0177)		(0.0176)	(0.0177)
Collab. within Africa	0.0976^***^	0.0963^***^	0.0974^***^	0.0974^***^	0.0969^***^	0.0983^***^		0.0969^***^
	(0.0237)	(0.0237)	(0.0237)	(0.0237)	(0.0237)	(0.0237)		(0.0237)
Collab. outside Africa	0.0686^***^	0.0684^***^	0.0683^***^	0.0684^***^	0.0687^***^	0.0675^***^	0.0661^***^	
	(0.0176)	(0.0176)	(0.0177)	(0.0177)	(0.0177)	(0.0177)	(0.0177)	
NOT South Africa x STEM ^c^	0.2022^***^	0.2003^***^	0.1992^***^	0.1977^***^	0.1992^***^	0.1993^***^	0.2000^***^	0.1984^***^
	(0.0278)	(0.0277)	(0.0277)	(0.0278)	(0.0277)	(0.0277)	(0.0277)	(0.0277)
NOT South Africa x Health ^c^	0.3184^***^	0.3166^***^	0.3154^***^	0.3147^***^	0.3157^***^	0.3151^***^	0.3153^***^	0.3153^***^
	(0.0313)	(0.0313)	(0.0313)	(0.0313)	(0.0313)	(0.0313)	(0.0312)	(0.0313)
NOT South Africa x SSH ^c^	0.1327^***^	0.1310^***^	0.1299^***^	0.1290^***^	0.1306^***^	0.1302^***^	0.1314^***^	0.1291^***^
	(0.0334)	(0.0334)	(0.0335)	(0.0335)	(0.0334)	(0.0334)	(0.0334)	(0.0334)
South Africa x STEM ^c^	0.0781^**^	0.0790^***^	0.0760^**^	0.0748^**^	0.0760^**^	0.0747^**^	0.0760^**^	0.0756^**^
	(0.0304)	(0.0304)	(0.0304)	(0.0304)	(0.0304)	(0.0304)	(0.0303)	(0.0304)
South Africa x Health ^c^	0.1531^***^	0.1523^***^	0.1514^***^	0.1515^***^	0.1511^***^	0.1490^***^	0.1488^***^	0.1493^***^
	(0.0376)	(0.0376)	(0.0376)	(0.0376)	(0.0376)	(0.0376)	(0.0376)	(0.0376)
Man x NOT Main care & housework	Omitted							
Man x Main care & housework	−0.0142							
	(0.0202)							
Woman x NOT Main care & housework	−0.0799^***^							
	(0.0280)							
Woman x Main care & housework	−0.1623^***^							
	(0.0213)							
Man x Early–career researcher		−0.1260^***^						
		(0.0241)						
Man x Mid–career researcher		0.0040						
		(0.0229)						
Man x Late–career researcher		Omitted						
Woman x Early–career researcher		−0.2696^***^						
		(0.0296)						
Woman x Mid–career researcher		−0.1340^***^						
		(0.0297)						
Woman x Late–career researcher		−0.0686^**^						
		(0.0319)						
Man x NO Study–related mobility			0.0375^*^					
			(0.0195)					
Man x Study–related mobility			Omitted					
Woman x NO Study–related mobility			−0.0861^***^					
			(0.0245)					
Woman x Study–related mobility			−0.1092^***^					
			(0.0316)					
Man x NO Work–related mobility				−0.0103				
				(0.0201)				
Man x Work–related mobility				Omitted				
Woman x NO Work–related mobility				−0.1434^***^				
				(0.0248)				
Woman x Work–related mobility				−0.0863^***^				
				(0.0321)				
Man x NO Collab. with own inst.					−0.0966^***^			
					(0.0201)			
Man x Collab. with own inst.					Omitted			
Woman x NO Collab. with own inst.					−0.2060^***^			
					(0.0265)			
Woman x Collab. with own inst.					−0.1260^***^			
					(0.0234)			
Man x NO Collab. with own country						−0.0712^***^		
						(0.0206)		
Man x Collab. with own country						Omitted		
Woman x NO Collab. with own country						−0.2094^***^		
						(0.0256)		
Woman x Collab. with own country						−0.0810^***^		
						(0.0302)		
Man x NO Collab. within Africa							−0.0623^**^	
							(0.0267)	
Man x Collab. within Africa							Omitted	
Woman x NO Collab. within Africa							−0.1998^***^	
							(0.0306)	
Woman x Collab. within Africa							0.0047	
							(0.0473)	
Man x NO Collab. outside Africa								−0.0527^***^
								(0.0204)
Man x Collab. outside Africa								Omitted
Woman x NO Collab. outside Africa								−0.1912^***^
								(0.0257)
Woman x Collab. outside Africa								−0.0850^***^
								(0.0291)
Constant	1.4537^***^	1.4499^***^	1.4252^***^	1.4766^***^	1.5545^***^	1.5362^***^	1.5284^***^	1.5220^***^
	(0.0627)	(0.0628)	(0.0640)	(0.0654)	(0.0642)	(0.0655)	(0.0681)	(0.0645)
Number of observations	4,676	4,676	4,676	4,676	4,676	4,676	4,676	4,676
F	53.3370	51.2079	53.1569	53.2326	53.1613	53.2852	53.5793	53.2730
R^2^	0.2158	0.2159	0.2153	0.2155	0.2153	0.2157	0.2166	0.2156
Adjusted R^2^	0.2118	0.2117	0.2112	0.2114	0.2112	0.2116	0.2126	0.2116
Log-likelihood	−3,654.85	−3,654.70	−3,656.56	−3,655.84	−3,656.51	−3,655.34	−36,52.56	−3,655.46

***, **, * Represent significance at the 0.01, 0.05 and 0.1 levels; Standard errors in parentheses.

x Represents the interaction between two variables.

a Late-career researcher is the omitted career-stage variable.

b All continuous variables were transformed using the natural logarithm: ln (*variable* + 1).

c South Africa x SSH fields is the omitted country x disciplinary field interactive variable.

### Age and gender

Table 1 shows the negative effect of gender on scientific publication output. In a linear academic-career model, namely continuous publication with regular and steady promotion up the organizational hierarchy, these characterizations reinforce the general perception that women are less capable researchers than their male colleagues. For instance, our results show that a greater proportion (≥50%) of care-work, family commitments and housework in the household have a general negative effect on the number of scientific papesr. The descriptive statistics showed that these chores are overwhelmingly performed by women. Regression 10 (Table 2) clearly shows that when women are responsible for the majority of the care- and housework, their scientific publication output suffers more than that of men who perform the majority of these chores, but also compared to other women who do not carry the main burden of care and household work^9^.

Our results also show that the less experienced (early-career) researchers publish less compared to their older colleagues, whether in mid- or late-career stage. This situation is exacerbated when we distinguish men and women according their career stage (based on chronological age) in the regression analysis (see regression 11 in Table 2). Early-career women are much more penalized than their male counterparts and compared to their older colleagues, whether male or female. Taking career stage as a proxy of maturity, the results in Table 2 show that the negative effect of being an early-career male researcher is almost equal to the negative effect of being a mid-career female researcher. These results clearly show that women “catch up” later in their careers, and that the difficulties posed by balancing a career with family responsibilities tend to be limited to the stage when children are relatively young. However, they ultimately cause women to be characterized as “slow” or “late” starters, and late achievers, in academia (Prozesky, 2008).

### Workload

Surprisingly, devoting time to research only has a weakly significant relationship with scholarly publications. It is important to stress that not all research leads to publications. More applied research may have a strong impact in a community, but not be published in an academic journal. The weak significance of the coefficient may reflect this fact. It is the number of hours spent supervising students that pays dividends in terms of scholarly publication output, hence highlighting the important role of graduate students in this regard (Larivière, 2012). Investing the same amount of supervising time with their graduate students as men do is slightly more beneficial for women (see regression 4 in Table 1), and so is devoting more time to research (see regression 5 in Table 1).

**Figure 1:**
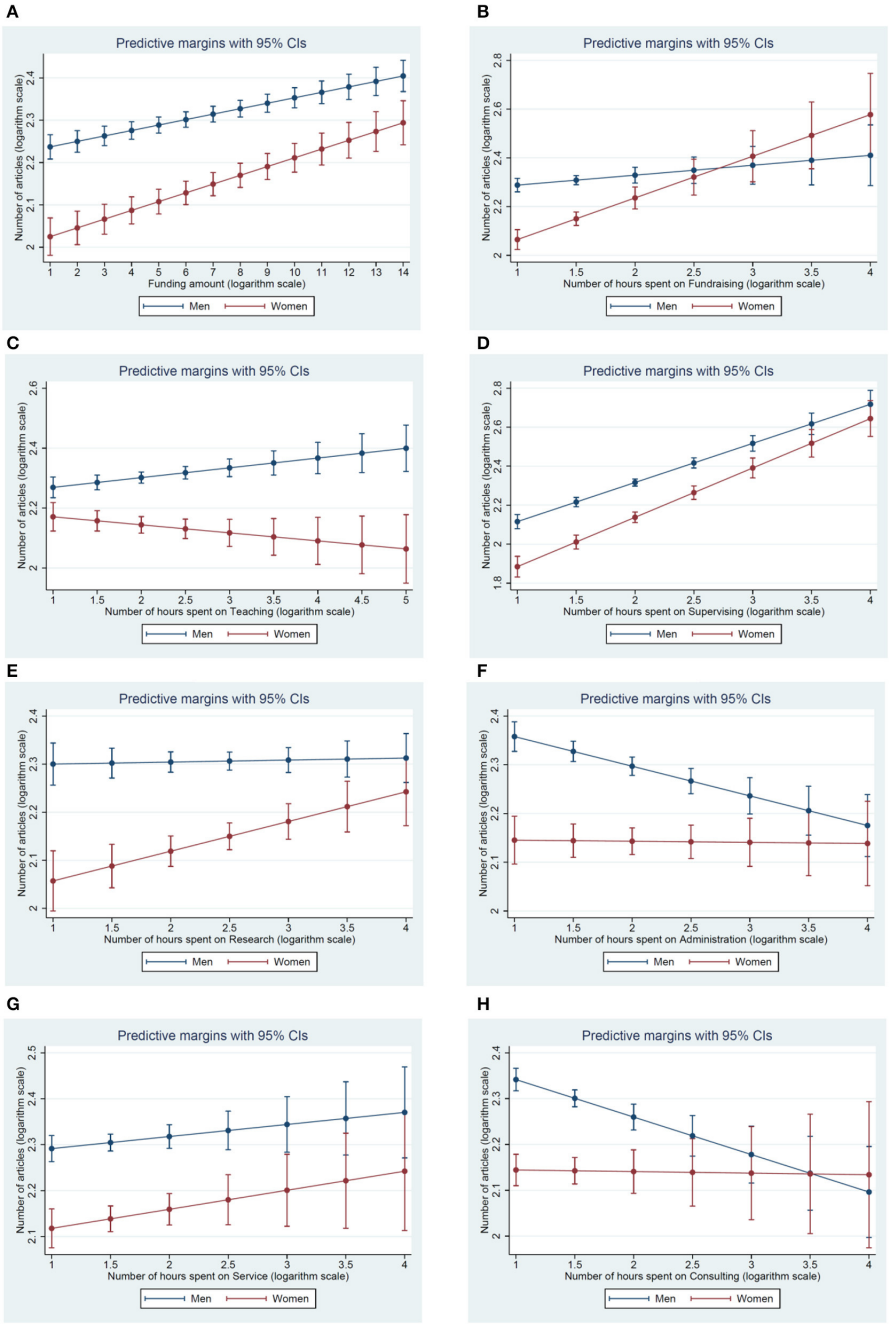
Predicted gender-differentiated scientific publication output by academic task. **(A)** Funding amount; **(B)** Fundraising time; **(C)** Teaching time; **(D)** Supervising time; **(E)** Research time; **(F)** Administrative time; **(G)** Service time; and **(H)** Consultation time.

Administrative duties and consultancy work are negatively associated with the number of articles published, but the effect is slightly less detrimental to women's scientific publication output (see regressions 6 and 8, respectively, in Table 1, as well as graphs *f* and *h* of Figure 1^10^, which illustrate the gender differences attributable to the regression results presented in Table 1). In other words, although the overall number of publications is still smaller for women, when they devote the same number of hours as men to either administration or consultation, the negative impact of such activities on their scientific publication output is partially mitigated.

Where women clearly suffer compared to their male colleagues is in regard to how detrimental to their publication record their teaching duties are. The coefficient of the variable counting the number of hours devoted to teaching is generally negative. But when in interaction with gender, the negative impact of a greater number of teaching hours on the scientific publication output of women is clear (see regression 3 in Table 1), and is the only negative effect of the time dedicated to various academic tasks. When men spend more time in the lecture hall, their scientific publication output increases while that of women diminishes (see graph *c* of Figure 1).

With the exception of teaching tasks, investing additional time on supervision, research, administrative duties, consultation, and fundraising results in more publications for women, even more so than for men. In other words, with an equal number of hours devoted to these facets of the academic profession, women publish more (see Figure 1). This finding highlights the scientific productivity women—all things being equal, women publish more per hour devoted to these academic tasks. The problem of women's lower publication output lies elsewhere, and is in essence related to work-family balance, as time dedicated to care- and other housework is competing with that devoted to academic tasks.

Before moving on to other factors, a comment about the number of hours dedicated to service is necessary. In general, the coefficient of the variable is weakly positive and significant in Tables 1 and 2, and no gender difference is found. In defining service as anything from counseling of patients, voluntary service within or outside the organization, to article review and editorial duties (the latter clearly indicating an active publishing record), we may have too wide a net to capture activities that would normally detract from publishing. This is a limit of the measure that we cannot overcome.

### Funding

Another crucial factor that positively contributes to scientific publication output is the amount of funds at the disposal of researchers. The amount of research funding raised for research purposes positively correlates with the number of articles published or accepted for publication. When women are as well funded as their male colleagues, they publish slightly more than them, but the effect is only weakly significant (see regression 2 in Table 2). In other words, when women have access to the same resources in terms of research funding, the gender gap disappears, *ceteris paribus*. Hence, our results do not give credence to the hypothesis that African women are less productive because of a lack of funding. Compared to men, devoting time to fundraising has a stronger positive relationship with increased scientific publication output (see regression 9 in Table 1).

### Mobility and collaboration

Contrarily to what common wisdom would suggest, researcher mobility (either during higher education or during paid work/employment) is not associated with a greater publication output (see Tables 1, 2). In all sets of regressions, study-related mobility^11^ exhibits a negative and significant coefficient, which may at first appear counterintuitive. This may be attributable to the fact that mobile researchers have already had the ability to publish, or may have been mobile prior to the 3 years on which the respondents were asked to report, or they may have had difficulty to adapt to, or reintegrate into, the local science system. The literature on migration suggests that economic factors play an important role in residency decision making (see for instance Prozesky and Beaudry, 2019). Considering the opportunities offered in the post-study period, for instance in finding positions abroad, some researchers from low- and middle-income countries (including countries in Africa) may decide not to return to their home countries. Our survey data do not allow us to disentangle the underlying reasons for these results. There are some weak gender effects (as shown in regression 12 in Table 2), but only for men, as shown in the coefficient comparison test presented in Table 9 in the Appendix.

A similar result is found for work-related mobility^12^, with the non-significance of the coefficient hiding weak gender effects (in regression 13 in Table 2), this time in favor of women: while no significant difference is noted between mobile and non-mobile men, women partially mitigate the gender effect by being mobile (the difference is weakly significant as shown in Table 9 in the Appendix).

More frequent research collaboration with colleagues is associated with more publications, and this holds true regardless of whether these collaborators are local or international. Understandably, local collaboration is more frequent compared with cooperating outside one's own country of residence (see Table 5 in the Appendix). The impact of the four dummy variables should therefore be considered as cumulative, i.e., the more frequently a researcher collaborates with a variety of colleagues located far and abroad, the higher the number of scientific publications. Once again, there are strong gender effects in the correlation between collaboration and scientific publication output, with women benefitting more than men from such relationships (the difference between the coefficients of the interactive variables is always greater for women). Regardless, collaborating is always a better decision—in terms of increasing the number of publications—for both genders (see regressions 14, 15, 16, and 17 in Table 2).

## Discussion

The present study is the first of its kind in Africa, as it attempts to shed light on the most important factors that influence the number of scientific articles published by both men and women academics on the continent (all countries in Africa were surveyed with the exception of Libya). The fact that this is a poorly researched topic in Africa is a point of concern if one considers Africa's extremely low scientific publication output (Beaudry and Mouton, 2018). Globally, very few studies on gender differences in publication have considered as many determinants as we have. Examples of studies that found women not to be significantly less productive than men when a variety of variables are controlled for, include those by Xie and Shauman (1998), D'amico et al. (2011), Bentley (2012), Frandsen et al. (2015), and Van Den Besselaar and Sandström (2017). These variables include individual background or personal characteristics (such as marital status, discipline, time lag between bachelor's and doctoral degrees, and years of experience beyond doctoral degree), and structural locations and resources (e.g., type of current institution, academic rank, teaching hours, research funding, and research assistance). It has long been suggested that gender differences in scientific publication output found in earlier studies may have resulted from omitting such variables. Our study contributes to the small body of scholarship that tries to remedy this.

In addition, most of those studies have been conducted in developed countries. As Teodorescu (2000, p. 219) already warned more than two decades ago, there is “a potential danger in applying the findings of Western literature on publication productivity to another national context”. Thus, it stands to reason that those important correlates explaining gender differences in scientific publication output may also differ between more- and less-developed countries. However, our results concur with similar attempts in developed countries to consider covariates related to publication and gender. African women are generally as prolific as their male counterparts when given the same opportunities.

We find that women may even produce more articles with the same time allocation on most academic tasks, implying that women's scientific publication output is at least on par with that of men. In addition, we found no discrepancies regarding funding: if endowed with research funding equal to that of men, women will produce more publications than them. This aligns with Beaudry and Larivière (2016), who found that women are more productive than men when provided with equal funding.

However, we found that childbearing and the ensuing care-work and housework hinder women's scientific publication output in Africa. More importantly, once shared between genders, childcare and housework have an equal impact on the number of scientific publications of both men and women. Our analysis corresponds to the small but growing body of existing literature on women scientists in Africa, which argues that these women contend with “pro-natalist cultures” that expect them to marry and have children (Tsikata, 2007), and that these reproductive responsibilities make it very hard for them to compete on equal terms with men (Mama, 2003), especially because of a traditional gendered division of labor within households (Tamale and Oloka-Onyango, 1997; Tettey, 2010; Akinsanya, 2012; Ben Hassine, 2014; Arthur and Arthur, 2016).

## Conclusion

Overall, the extent to which gender mediates the relationship between a range of variables and scientific publication output in Africa is undeniable. This leads us to call for a change in how academic career success is conceptualized. The traditional and still predominant preoccupation with constant (and increasing) productivity throughout one's career assumes a masculine life cycle. It does not consider that women's careers are more “fractured” or discontinuous because they shape their professional lives in relation to the lives of partners, elderly parents and children. And, for some determinants of scientific publication output, women outperform men when input and resources are equal, implying women's capability.

During life-story interviews with faculty at the University of Ghana, women themselves described the “stagnation of their research and writing during their intensive childbearing and rearing years” (Tsikata, 2007, p. 35). The effect is also indirect: mobility and collaboration, which we found offset the effect of gender on scientific publication output, are closely related to childbearing, care-work and housework. It has often been noted that many women scientists are limited in their geographic mobility by family demands. In Africa, female researchers have reported difficulties traveling to conferences, for example, because they assume they are the primary domestic caregiver at home, thereby restricting their professional networks and collaboration opportunities (Campion and Shrum, 2004; Akinsanya, 2012).

Women are expected to contribute to research as much as their male counterparts, while still fulfilling their domestic roles and continuing to be the family's primary caregivers. These expectations are particularly strong in developing regions but are generally not considered by universities, the primary research institutions in African countries, when academics are promoted. Instead, “the academy judges women at par with men when considering their output and competence” (Tamale and Oloka-Onyango, 1997, p. 20, Tsikata, 2007). Furthermore, many academic institutions do not provide any allowances in their policies or employment contracts for women in the role of caregiver (e.g., day-care facilities or family-responsibility leave). As long as scientific publication output is measured and rewarded in ways that ignore these gender differences, women scientists in Africa will continue to be judged and treated as the “less productive” gender.

Not only do academic institutions need to change, but the very fabric of our society also requires transformation, which should primarily be brought about by a drastic modification of how male children are socialized, to enable them, as adults, to take on an equal or even primary share of domestic and parenting responsibilities. From a broader societal perspective, we call for a change in focus away from the socialization of young women, which may render them ill-prepared for traditionally male workplace culture and values, to a focus on the socialization of male children, which severely limits their potential to fulfill caregiver roles in our society. Women who work in science have long challenged the limits of their socialized gender roles by rejecting, for example, the norm that their maternal role should be their primary commitment. We therefore should not seek the solution among those women or even girl children, but rather in the institutions of education and the family, which still do not normatively prescribe men's equal contribution to household chores and care-work, especially in African countries (Stromquist, 2007; Morrell et al., 2012). Unpaid work and its effects on academic careers should cease to be defined as a “women's issue” for which women, rather than their male colleagues or partners, have to take primary responsibility.

Such modifications in norms and behavior would only be established and maintained, however, if reinforced by the institutions of work, through the provision of equal maternity and paternity leave, allowances for teaching hours to be in sync with crèche/school times, and travel support for parents with young children, regardless of the gender of the parent, to attend conferences or for short research stays. A historical perspective reminds us that changes in the extent of support offered to mothers in the academic work environment, and changes in values related to women's role in society, are possible. Women have come a long way in negotiating workplace support, from when maternity leave was not even an option and when raising the issue of childcare responsibilities at work was considered taboo, to a more adapting and flexible working environment for mothers in academia. Generational differences, and therefore changes over time, are also becoming apparent concerning domestic arrangements, as co-parenting is increasingly less likely to represent a violation of societal norms (Pattnaik and Srirarm, 2010).

However, academic work still assumes support in the domestic sphere. As we show, this assumption has become not only an empirically untenable stereotype, but one that maintains unfair assessments of women's scientific abilities and precludes the optimal use of the skilled human resources at our disposable.

## Limitations

The research presented in this article is not free from limitations. First, the list of emails used to solicit authors was predominantly based on Clarivate™ Web of Science, although several other sources were used to complement the information. This method will have undoubtedly led to an underestimation of social scientists and humanities scholars, especially in the disciplines where monographs are the preferred publication output. In addition to the relatively low resulting sample size (which we estimate to about 10% of African researchers), it is possible that our researchers were not reached. The validity of abovementioned interpretations hence need to be put into this perspective. Second, the dependent variable used in the regression analysis is a self-reported measure of the number of peer-reviewed articles published. Individuals may have miscounted and/or included publications that had not been peer-reviewed. Third, it would have been important to disentangle gender effects within the co-authorship list (single authors, mix of men and women in the list of coauthors, etc.,) to measure the correlation between (1) research collaboration between opposite/same gender, (2) scientific publication output, and (3) impact of the papers published. Unfortunately, the survey design does not allow us to disentangle the number of single-author from the mixed-gender multiple-authors publications, which is an important limitation to our study. As mentioned, the survey asked respondents to only provide the number of publications, not the citations or other metadata related to the publications, hence the authorship composition of the publications is not available. We are nevertheless confident that the results presented are of value to the African research community.

## Data availability statement

The datasets presented in this article are not readily available because sharing the data is restricted by both the Polytechnique Montreal and Stellenbosch Ethics Boards due to the consent given by the participants. Requests to access the datasets should be directed to CB, catherine.beaudry@polymtl.ca.

## Ethics statement

The studies involving human participants were reviewed and approved by the Ethics Boards of Polytechnique Montréal (N/Réf: CÉR-1516-43) and Stellenbosch University (Proposal #: SU HSD-002130). The participants provided their written informed consent to participate in this study.

## Author contributions

Literature search: CB (30%)—HP (60%)—CS-P (0%)—SM (10%). Study design: CB (50%)—HP (40%)—CS-P (10%)—SM (0%). Data collection: CB (50%)—HP (50%)—CS-P (0%)—SM (0%). Data analysis: CB (40%)—HP (20%)—CS-P (30%)—SM (10%). Data interpretation: CB (40%)—HP (20%)—CS-P (20%)—SM (20%). Writing: CB (50%)—HP (30%)—CS-P (0%)—SM (20%). All authors contributed to the article and approved the submitted version.

## Footnotes

^1^Mouton (2019) lists some of the tasks performed by SGCs as providing research grants, scholarships and loans, funding infrastructure, supporting scientific publishing, providing science advocacy and advice, collecting data, setting research priorities, fostering and maintaining collaboration, as well as coordinating the innovation system.

^2^The full list of countries covered by the survey is as follows: Algeria, Angola, Benin, Botswana, Burkina Faso, Burundi, Cameroon, Cape Verde, Central African Republic, Chad, Comoros, Congo, Congo People's Republic, Côte d'Ivoire, Democratic Republic of Congo, Djibouti, Egypt, Equatorial Guinea, Eritrea, Ethiopia, Gabon, Gambia, Ghana, Guinea, Guinea Bissau, Kenya, Lesotho, Liberia, Libya, Madagascar, Malawi, Mali, Mauritania, Mauritius, Morocco, Mozambique, Namibia, Niger, Nigeria, Reunion, Rwanda, Sao Tome & Principe, Senegal, Seychelles, Sierra Leone, Somalia, South Africa, South Sudan, Sudan, Swaziland, Tanzania, Togo, Tunisia, Uganda, Western Sahara, Zambia, Zimbabwe. Libya was excluded from the survey as the political situation was too unstable at the time.

^3^A list of emails for the authors for which an African affiliation was listed on a publication in the Web of Science was complemented with other data sources, including the South African Knowledgebase database. Duplicate and inactive emails were eliminated. A detailed account of the survey methodology is provided in Appendix 1 (p. 183) the free online book: The next Generation of Scientists in Africa: https://www.africanminds.co.za/the-next-generation-of-scientists/.

^4^In one of the survey questions, the respondents were asked to specify the sector of employment of their current job. Because the survey did not specify when they started their employment or whether they recently changed jobs, we kept all observations. The resulting sample is composed of: 3,406 in higher/tertiary education; 576 in public research institutions; 80 in private research institutions; 157 in business enterprises; 159 in non-governmental/non-profit organizations; and 298 in other organizations. To further justify our decision, the majority of individuals in all these categories, except other organizations, teach.

^5^We used the Leiden subject categories (https://www.leidenranking.com/Content/CWTS%20Leiden%20Ranking%202022%20-%20Main%20fields.xlsx), which were mapped into STEM, Health Science and SSH. Biomedical and Health Sciences was classified as Health Sciences, Social Sciences and Humanities as SSH, and the other three categories were aggregated into STEM.

^6^During the course of the research, we estimated Poisson and negative binomial regressions on the raw variable (the number of articles that is a count variable) as well as ordinary least squares (OLS) on the log-transformed variable. Only the latter is presented in this paper as it provides the most robust results.

^7^During the course of the research, we tested both career age (number of years since highest degree) and chronological age. There were strong cohort effects with the former (the older generation having obtained their PhDs at a later age than the younger generation). Results were more robust with the use of real age, which was separated into three groups: early career ( ≤ 40 years of age), mid-career (>40 and ≤ 50 years of age), and late career (>50 years of age).

^8^Individuals were classified according to the discipline of their highest qualification.

^9^Tables 7–10 in the Appendix present the results of the 2 by 2 tests between the coefficients of the gender-interactive variables from the regression results in Tables 1 and 2.

^10^Figure 1 plots the predictive margins for the effect of each of the eight continuous variables on the dependent variable.

^11^Study-related mobility during higher education implies that the respondents obtained their highest degree from an institution in a country other than what they would consider their home country.

^12^Work-related mobility during paid work/employment implies that the respondent has worked in a country other than what he/she would consider his/her home country.
