# Supplementary material for: Factors that affect scientific publication in Africa—A gender perspective
Source: Front Res Metr Anal. 2023 Feb 20;8:1040823. doi: 10.3389/frma.2023.1040823 (PMC9986590; doi:10.3389/frma.2023.1040823)
Supplement: Supplementary file 1 [file Data_Sheet_1.docx]

# Appendix

Table 3 Sample size per country

| **Name of Country** | **Number of respondents^a^** | **Name of Country** | **Number of respondents^a^** |
| --- | --- | --- | --- |
| South Africa | 1,721 | Burkina Faso | 64 |
| Nigeria | 660 | Côte d’Ivoire | 61 |
| Algeria | 286 | Botswana | 60 |
| Tunisia | 275 | Senegal | 57 |
| Kenya | 186 | Benin | 45 |
| Morocco | 170 | Malawi | 37 |
| Egypt | 149 | Congo | 24 |
| Ethiopia | 143 | Togo | 20 |
| Uganda | 133 | Madagascar | 18 |
| Cameroon | 123 | Niger | 16 |
| Ghana | 117 | Mali | 15 |
| Zambia | 91 | Democratic Republic of the Congo | 14 |
| Zimbabwe | 88 | Gabon | 10 |
| Tanzania | 78 | Central African Republic, Guinea, Burundi, Seychelles, and Chad^b^ | 15 |
| **Total** |  |  | **4,676** |

Note: ^a^ The number of respondents by country is provided in appendix 1 (pp. 184-185) of the published book: The next Generation of Scientists in Africa: <https://www.africanminds.co.za/the-next-generation-of-scientists/>.
^b^ For confidentiality reasons, and to respect the ethics certificate of this project, data where the number of respondents is less than 10 cannot be disclosed.

Table 4 Variable description

| **Variable** | **Type** | **Description** | **Survey questions^c^** |
| --- | --- | --- | --- |
| **Dependent variable** | | | |
| **Number of articles** | Continuous | Natural logarithm of the number of articles published/accepted (including co-authored) in refereed or peer-reviewed academic journals | “*Please indicate how many of the following research output types you have produced over the last three years: articles published/accepted (including co-authored) in refereed or peer-reviewed academic journals*” |
| **Independent variables** | | | |
| **Main care & housework** | Dummy | Takes the value 1 if the proportion of care-work and general housework for all dependents performed by the respondent is above 50% of the total, and 0 otherwise | First: “*How is the care-work for all dependents distributed in your family or relationship? (in percentage)* *[ ]% me [ ]% partner [ ]% others (e.g. extended family, paid service)*”  Second: “*How is general housework distributed in your family or relationship? (in percentage) [ ]% me [ ]% partner [ ]% others (e.g. paid service)*” |
| **Early-career researcher** | Dummy | Takes the value 1 if the respondent is an early-career researcher (≤ 40 years of age), and 0 otherwise | “*What is your year of birth?*” |
| **Mid-career researcher** | Dummy | Takes the value 1 if the respondent is a mid-career researcher (< 40 and ≥ 50 years of age), and 0 otherwise |  |
| **Late-career researcher** **(reference category)** | Dummy | Takes the value 1 if the respondent is a late-career researcher (< 50 years of age), and 0 otherwise |  |
| **Teaching hours** | Continuous^a^ | Number of working hours spent on undergraduate and postgraduate teaching | First “*On average, how many hours do you spend on your main job per week?*”  Combined with second “*In a typical year, what percentage of your working time do you spend on each of the following tasks?*” |
| **Supervising hours** | Continuous^a^ | Number of working hours spent on training/supervising postgraduate students |  |
| **Research hours** | Continuous^a^ | Number of working hours spent on research |  |
| **Admin hours** | Continuous^a^ | Number of working hours spent on administration and management |  |
| **Service hours** | Continuous^a^ | Number of working hours spent on service (counselling of patients, voluntary services within or outside the organisation, article review, editorial duties) |  |
| **Consultation hours** | Continuous^a^ | Number of working hours spent on consultancy |  |
| **Fundraising hours** | Continuous^a^ | Number of working hours spent on raising funds/grants for research |  |
| **Funding** | Continuous | Total amount of research funding received during the past three years | “*Which amount best correspond to the total amount of research funding you have received during the past three years?*” |
| **Study-related mobility** | Dummy | Takes the value 1 if the respondent obtained his/her highest degree from an institution in a country other than the country of his/her home country, and 0 otherwise | “*Did you complete all aspects of your doctoral (or equivalent) education in what you would consider to be your home country?*” |
| **Work-related mobility** | Dummy | Takes the value 1 if the respondent has worked in a country other than what he/she would consider his/her home country (i.e. abroad), and 0 otherwise | “*During the past five years, have you lived or worked in a country other than what you would consider your home country?*” |
| **Collaboration with  own inst./country** | Dummy^b^ | Takes the value 1 if the respondent has collaborated often (4) or very often/always (5) with researchers from his/her own institution, and 0 otherwise | “*How often during your career so far have you collaborated in joint research projects with the following groups of researchers?*” |
| **Collaboration with own country** | Dummy^b^ | Takes the value 1 if the respondent has collaborated often (4) or very often/always (5) with researchers from his/her own country, and 0 otherwise |  |
| **Collaboration within Africa** | Dummy^b^ | Takes the value 1 if the respondent has collaborated often (4) or very often/always (5) with researchers from institutions in **other** African countries, and 0 otherwise |  |
| **Collaboration outside Africa** | Dummy^b^ | Takes the value 1 if the respondent has collaborated often (4) or very often/always (5) with researchers institutions outside of Africa (e.g. Europe, North America, Asia, etc.), and 0 otherwise |  |
| **Moderating variable** | | | |
| **Woman** | Dummy | Takes the value 1 if the respondent is a woman, and 0 otherwise | “*Are you: [ ] Male [ ] Female?*” |
| **Control variables** | | | |
| **South Africa** | Dummy | Takes the value 1 if the respondent’s country of work is South Africa, and 0 otherwise | “*In which country do you currently work / reside?*” |
| **STEM fields** | Dummy | Takes the value 1 if the respondent’s research discipline is in Science, Technology, Engineering and Mathematics (STEM) fields, and 0 otherwise | “*In which field did you obtain your highest qualification?*” |
| **Health fields** | Dummy | Takes the value 1 if the respondent’s research discipline is in the Health Science fields, and 0 otherwise |  |
| **SSH fields** | Dummy | Takes the value 1 if the respondent’s research discipline is in the Social Sciences and Humanities fields, and 0 otherwise |  |

Note: ^a^ The number of hours was calculated by multiplying the total number of hours reported by the respondent and the corresponding proportion of their time devoted to a particular academic task.
^b^ 5-point Likert scales (1 = Never or very rarely, 2 = Rarely, 3 = Sometimes, 4 = Often, 5 = Very often/always) transformed into dummy variables.
^c^ A slightly modified version of the questionnaire used for this study is provided in appendix 2 of the published book: The next Generation of Scientists in Africa: <https://www.africanminds.co.za/the-next-generation-of-scientists/>.

Table 5 Descriptive statistics

|  | **All** | | | |  | **Men** | |  | **Women** | | **Diff.** |
| --- | --- | --- | --- | --- | --- | --- | --- | --- | --- | --- | --- |
| **Variables** | **Mean** | **Std. Dev.** | **min** | **max** |  | **Mean** | **Std. Dev.** |  | **Mean** | **Std. Dev.** | **p** |
| **Dependent variable** | | | | | | | | | | | |
| ***Number of articles*** | 8.3488 | (6.6592) | 0 | 21 |  | 8.8994 | (6.7961) |  | 7.1065 | (6.1634) | *** |
| **Independent variables** | | | | | | | | | | | |
| ***Age* ^a^** | 46.1642 | (10.1235) | 23 | 75 |  | 46.6401 | (10.2612) |  | 45.0905 | (9.7241) |  |
| ***Early-career researcher*** | 0.3317 | (0.4709) | 0 | 1 |  | 0.3139 | (0.4641) |  | 0.3719 | (0.4835) | *** |
| ***Mid-career researcher*** | 0.3518 | (0.4776) | 0 | 1 |  | 0.3556 | (0.4788) |  | 0.3433 | (0.4750) |  |
| ***Late-career researcher*** | 0.3165 | (0.4652) | 0 | 1 |  | 0.3306 | (0.4705) |  | 0.2848 | (0.4515) | *** |
| ***Proportion of care- & housework* ^a^** | 43.6276 | (26.5533) | 0 | 100 |  | 37.4565 | (24.6117) |  | 57.5513 | (25.4914) | *** |
| ***Main care & housework*** | 0.4279 | (0.4948) | 0 | 1 |  | 0.3164 | (0.4651) |  | 0.6797 | (0.4668) | *** |
| ***Teaching hours*** | 7.8602 | (8.4573) | 0 | 69.7 |  | 8.1059 | (8.6217) |  | 7.3057 | (8.0496) | *** |
| ***Supervising hours*** | 5.7495 | (5.8676) | 0 | 50.0 |  | 5.4726 | (5.5534) |  | 6.3743 | (6.4797) | *** |
| ***Research hours*** | 10.5896 | (10.2197) | 0 | 90.0 |  | 10.4045 | (9.9809) |  | 11.0071 | (10.7309) | * |
| ***Admin hours*** | 5.7383 | (7.7908) | 0 | 68.8 |  | 5.3547 | (7.6133) |  | 6.6038 | (8.1135) | *** |
| ***Service hours*** | 2.6186 | (4.6384) | 0 | 80.0 |  | 2.4549 | (4.4431) |  | 2.9878 | (5.0335) | *** |
| ***Consultation hours*** | 1.8720 | (4.8592) | 0 | 100.0 |  | 2.0049 | (4.9406) |  | 1.5722 | (4.6581) | *** |
| ***Fundraising hours*** | 1.7458 | (2.9381) | 0 | 33.3 |  | 1.7001 | (2.9201) |  | 1.8488 | (2.9768) |  |
| ***Funding*** | 82,489 | (208226) | 0 | 1,000,000 |  | 80,634 | (207475) |  | 86,675 | (209921) |  |
| ***Study-related mobility*** | 0.3606 | (0.4802) | 0 | 1 |  | 0.4012 | (0.4902) |  | 0.2688 | (0.4435) | *** |
| ***Work-related mobility*** | 0.3349 | (0.4720) | 0 | 1 |  | 0.3654 | (0.4816) |  | 0.2660 | (0.4420) | *** |
| ***Collaboration with own inst.*** | 0.6097 | (0.4879) | 0 | 1 |  | 0.6244 | (0.4844) |  | 0.5766 | (0.4943) | *** |
| ***Collaboration with own country*** | 0.3516 | (0.4775) | 0 | 1 |  | 0.3627 | (0.4808) |  | 0.3266 | (0.4691) | ** |
| ***Collaboration within Africa*** | 0.1518 | (0.3589) | 0 | 1 |  | 0.1660 | (0.3722) |  | 0.1198 | (0.3248) | *** |
| ***Collaboration outside Africa*** | 0.3749 | (0.4841) | 0 | 1 |  | 0.3830 | (0.4862) |  | 0.3565 | (0.4791) | * |
| **Moderating variable** | | | | | | | | | | | |
| ***Woman*** | 0.3071 | (0.4613) | 0 | 1 |  |  |  |  |  |  |  |
| **Independent variables** | | | | | | | | | | | |
| ***South Africa*** | 0.3482 | (0.4764) | 0 | 1 |  | 0.2728 | (0.4455) |  | 0.5181 | (0.4998) | *** |
| ***STEM fields*** | 0.5357 | (0.4988) | 0 | 1 |  | 0.5762 | (0.4942) |  | 0.4443 | (0.4971) | *** |
| ***Health fields*** | 0.2254 | (0.4179) | 0 | 1 |  | 0.2108 | (0.4079) |  | 0.2584 | (0.4379) | *** |
| ***SSH fields*** | 0.2389 | (0.4264) | 0 | 1 |  | 0.2130 | (0.4095) |  | 0.2974 | (0.4573) | *** |

Note: N_total_ = 4,676 observations; N_men_ = 3,240 observations; N_women_ = 1,436 observations.
^***, **, *^ represent significance at the 0.01, 0.05 and 0.1 levels.
^a^ Age and the Proportion of care- & housework were transformed into dummy variables prior to being used in the regression analysis.

Table 6 Correlation table

| **Variables** |  | **1** |  | **2** |  | **3** |  | **4** |  | **5** |  | **6** |  | **7** |  | **8** |  | **9** |  | **10** |  | **11** |  | **12** |  |  |
| --- | --- | --- | --- | --- | --- | --- | --- | --- | --- | --- | --- | --- | --- | --- | --- | --- | --- | --- | --- | --- | --- | --- | --- | --- | --- | --- |
| ***Number of articles* ^a^** | **1** | 1 |  |  |  |  |  |  |  |  |  |  |  |  |  |  |  |  |  |  |  |  |  |  |  |  |
| ***Woman*** | **2** | -0.1257 | * | 1 |  |  |  |  |  |  |  |  |  |  |  |  |  |  |  |  |  |  |  |  |  |  |
| ***Age* ^b^** | **3** | 0.1380 | * | -0.0706 | * | 1 |  |  |  |  |  |  |  |  |  |  |  |  |  |  |  |  |  |  |  |  |
| ***Early-career researcher*** | **4** | -0.1620 | * | 0.0568 | * | -0.7408 | * | 1 |  |  |  |  |  |  |  |  |  |  |  |  |  |  |  |  |  |  |
| ***Mid-career researcher*** | **5** | 0.0838 | * | -0.0118 |  | -0.0673 | * | -0.5190 | * | 1 |  |  |  |  |  |  |  |  |  |  |  |  |  |  |  |  |
| ***Late-career researcher*** | **6** | 0.0779 | * | -0.0454 | * | 0.8190 | * | -0.4794 | * | -0.5013 | * | 1 |  |  |  |  |  |  |  |  |  |  |  |  |  |  |
| ***Main care & housework*** | **7** | -0.1063 | * | 0.3387 | * | -0.0808 | * | 0.0810 | * | -0.0398 | * | -0.0412 | * | 1 |  |  |  |  |  |  |  |  |  |  |  |  |
| ***Teaching hours* ^a^** | **8** | 0.0889 | * | -0.0513 | * | -0.0162 |  | -0.0497 | * | 0.0623 | * | -0.0137 |  | -0.0019 |  | 1 |  |  |  |  |  |  |  |  |  |  |
| ***Supervising hours*** | **9** | 0.2876 | * | 0.0541 | * | 0.1578 | * | -0.1749 | * | 0.0642 | * | 0.1111 | * | -0.0186 |  | 0.3623 | * | 1 |  |  |  |  |  |  |  |  |
| ***Research hours* ^a^** | **10** | 0.1067 | * | 0.0159 |  | -0.0866 | * | 0.0991 | * | -0.0410 | * | -0.0582 | * | 0.0268 |  | -0.1163 | * | 0.1392 | * | 1 |  |  |  |  |  |  |
| ***Admin hours* ^a^** | **11** | -0.0090 |  | 0.0951 | * | 0.0645 | * | -0.0899 | * | 0.0167 |  | 0.0738 | * | 0.0186 |  | -0.0424 | * | 0.1356 | * | 0.0706 | * | 1 |  |  |  |  |
| ***Service hours* ^a^** | **12** | 0.0555 | * | 0.0593 | * | 0.0487 | * | -0.0516 | * | 0.0261 |  | 0.0254 |  | -0.0100 |  | 0.0227 |  | 0.1362 | * | 0.0961 | * | 0.2123 | * | 1 |  |  |
| ***Consultation hours* ^a^** | **13** | -0.0387 | * | -0.0846 | * | 0.0507 | * | -0.0335 | * | 0.0068 |  | 0.0270 |  | -0.0593 | * | -0.1107 | * | -0.0323 | * | -0.0185 |  | 0.0391 | * | 0.1372 | * |  |
| ***Fundraising hours* ^a^** | **14** | 0.1851 | * | 0.0269 |  | -0.0384 | * | 0.0160 |  | 0.0163 |  | -0.0329 | * | -0.0219 |  | -0.0896 | * | 0.1915 | * | 0.3075 | * | 0.2432 | * | 0.1478 | * |  |
| ***Funding* ^a^** | **15** | 0.2345 | * | 0.0357 | * | 0.1351 | * | -0.1222 | * | 0.0082 |  | 0.1153 | * | -0.0181 |  | -0.0403 | * | 0.2041 | * | 0.2321 | * | 0.1247 | * | 0.0095 |  |  |
| ***Study-related mobility*** | **16** | 0.0376 | * | -0.1272 | * | 0.0285 |  | -0.0371 | * | 0.0279 |  | 0.0090 |  | -0.0770 | * | 0.0095 |  | -0.0252 |  | 0.0020 |  | -0.0684 | * | -0.0519 | * |  |
| ***Work-related mobility*** | **17** | 0.0558 | * | -0.0972 | * | -0.1343 | * | 0.1180 | * | -0.0066 |  | -0.1127 | * | -0.0377 | * | -0.0169 |  | -0.0257 |  | 0.1080 | * | -0.0575 | * | -0.0009 |  |  |
| ***Collaboration with own inst.*** | **18** | 0.1770 | * | -0.0452 | * | -0.0072 |  | -0.0109 |  | 0.0147 |  | -0.0041 |  | -0.0567 | * | -0.0615 | * | 0.0782 | * | 0.1296 | * | 0.0121 |  | 0.0095 |  |  |
| ***Collaboration with own country*** | **19** | 0.1683 | * | -0.0348 | * | 0.0093 |  | -0.0260 |  | 0.0222 |  | 0.0035 |  | -0.0367 | * | -0.0859 | * | 0.0371 | * | 0.0973 | * | 0.0256 |  | -0.0009 |  |  |
| ***Collaboration within Africa*** | **20** | 0.1603 | * | -0.0595 | * | 0.0082 |  | -0.0323 | * | 0.0277 |  | 0.0042 |  | -0.0793 | * | -0.0882 | * | 0.0113 |  | 0.0769 | * | 0.0265 |  | -0.0255 |  |  |
| ***Collaboration outside Africa*** | **21** | 0.1663 | * | -0.0252 |  | 0.0097 |  | -0.0154 |  | 0.0114 |  | 0.0039 |  | -0.0251 |  | -0.0966 | * | 0.0763 | * | 0.1226 | * | -0.0014 |  | -0.0391 | * |  |
| ***South Africa*** | **22** | -0.0948 | * | 0.2375 | * | 0.1229 | * | -0.0276 |  | -0.0984 | * | 0.1291 | * | 0.1418 | * | -0.0773 | * | 0.1692 | * | 0.1258 | * | 0.2880 | * | 0.1185 | * |  |
| ***STEM fields*** | **23** | 0.0192 |  | -0.1220 | * | -0.0653 | * | 0.0447 | * | -0.0155 |  | -0.0294 | * | -0.0260 |  | -0.0067 |  | -0.0212 |  | 0.0058 |  | -0.1041 | * | -0.1810 | * |  |
| ***Health fields*** | **24** | 0.1054 | * | 0.0525 | * | -0.0128 |  | 0.0048 |  | 0.0270 |  | -0.0326 | * | -0.0238 |  | -0.0239 |  | 0.0200 |  | -0.0051 |  | 0.0397 | * | 0.2135 | * |  |
| ***SSH fields*** | **25** | -0.1258 | * | 0.0913 | * | 0.0889 | * | -0.0570 | * | -0.0084 |  | 0.0663 | * | 0.0537 | * | 0.0313 | * | 0.0051 |  | -0.0017 |  | 0.0828 | * | 0.0026 |  |  |
|  |  |  |  |  |  |  |  |  |  |  |  |  |  |  |  |  |  |  |  |  |  |  |  |  |  |  |
|  |  | **13** |  | **14** |  | **15** |  | **16** |  | **17** |  | **18** |  | **19** |  | **20** |  | **21** |  | **22** |  | **23** |  | **24** |  | **25** |
| ***Consultation hours* ^a^** | **13** | 1 |  |  |  |  |  |  |  |  |  |  |  |  |  |  |  |  |  |  |  |  |  |  |  |  |
| ***Fundraising hours* ^a^** | **14** | 0.0700 | * | 1 |  |  |  |  |  |  |  |  |  |  |  |  |  |  |  |  |  |  |  |  |  |  |
| ***Funding* ^a^** | **15** | -0.0397 | * | 0.3939 | * | 1 |  |  |  |  |  |  |  |  |  |  |  |  |  |  |  |  |  |  |  |  |
| ***Study-related mobility*** | **16** | 0.0168 |  | 0.0657 | * | 0.0908 | * | 1 |  |  |  |  |  |  |  |  |  |  |  |  |  |  |  |  |  |  |
| ***Work-related mobility*** | **17** | 0.0431 | * | 0.1273 | * | 0.0703 | * | 0.1598 | * | 1 |  |  |  |  |  |  |  |  |  |  |  |  |  |  |  |  |
| ***Collaboration with own inst.*** | **18** | -0.0020 |  | 0.1461 | * | 0.1472 | * | 0.0046 |  | -0.0203 |  | 1 |  |  |  |  |  |  |  |  |  |  |  |  |  |  |
| ***Collaboration with own country*** | **19** | 0.0230 |  | 0.1750 | * | 0.1399 | * | -0.0016 |  | 0.0260 |  | 0.2411 | * | 1 |  |  |  |  |  |  |  |  |  |  |  |  |
| ***Collaboration within Africa*** | **20** | 0.0087 |  | 0.1765 | * | 0.1703 | * | 0.1229 | * | 0.1366 | * | 0.1479 | * | 0.2776 | * | 1 |  |  |  |  |  |  |  |  |  |  |
| ***Collaboration outside Africa*** | **21** | -0.0306 | * | 0.2015 | * | 0.2740 | * | 0.1251 | * | 0.1525 | * | 0.0790 | * | 0.2171 | * | 0.2632 | * | 1 |  |  |  |  |  |  |  |  |
| ***South Africa*** | **22** | -0.0147 |  | 0.0854 | * | 0.1649 | * | -0.1963 | * | -0.1315 | * | -0.0595 | * | -0.0257 |  | -0.1016 | * | 0.0080 |  | 1 |  |  |  |  |  |  |
| ***STEM fields*** | **23** | -0.0541 | * | 0.0332 | * | -0.0105 |  | 0.0168 |  | -0.0117 |  | 0.0577 | * | 0.0739 | * | -0.0040 |  | 0.0655 | * | -0.1234 | * | 1 |  |  |  |  |
| ***Health fields*** | **24** | 0.0464 | * | 0.0197 |  | 0.0045 |  | -0.0288 | * | 0.0065 |  | 0.0749 | * | 0.0144 |  | 0.0313 | * | 0.0136 |  | -0.0483 | * | -0.5795 | * | 1 |  |  |
| ***SSH fields*** | **25** | 0.0178 |  | -0.0581 | * | 0.0079 |  | 0.0086 |  | 0.0073 |  | -0.1409 | * | -0.1005 | * | -0.0260 |  | -0.0899 | * | 0.1917 | * | -0.6018 | * | -0.3022 | * | 1 |

Note: Significance level: * p<0.05.
^a^ All continuous variables were transformed using the natural logarithm: ln(*variable* + 1);
^b^ Age is not used in the regression analysis.

Table 7 Interactive variable coefficient comparison tests (t-tests) – Gender vs Main care and housework

|  | | **Women** | |  |  | |
| --- | --- | --- | --- | --- | --- | --- |
|  |  | **Main care & housework** | |  |  |  |
| **Men** | **Main care & housework** | **NO** | **YES** |  | **Main care & housework** |  |
|  | **NO** | ******* | ******* |  | NO vs YES [Women] | *** |
|  | **YES** | ****** | ******* |  | NO vs YES [Men] | NS |

Notes: ^***, **, *^ represent significance at the 0.01, 0.05 and 0.1 levels.

Table 8 Interactive variable coefficient comparison tests (t-tests) – Gender vs career stage

|  | | | | | **Women** | | |
| --- | --- | --- | --- | --- | --- | --- | --- |
|  |  |  |  |  | Early- vs Mid-career | Early- vs Late-career | Mid- vs Late-career |
|  |  |  |  |  | *** | *** | * |
|  |  |  |  |  | **Women** | | |
|  |  |  |  |  | **Early-career** | **Mid-career** | **Late-career** |
| **Men** | Early- vs Mid-career | *** | **Men** | **Early-career** | *** | NS | * |
|  | Early- vs Late-career | *** |  | **Mid-career** | *** | *** | ** |
|  | Mid- vs Late-career | NS |  | **Late-career** | *** | *** | ** |

Notes: ^***, **, *^ represent significance at the 0.01, 0.05 and 0.1 levels.

Table 9 Interactive variable coefficient comparison tests (t-tests) – Gender vs mobility

| **a)** | | **Women** | |  |  | |
| --- | --- | --- | --- | --- | --- | --- |
|  |  | **Study-based mobility** | |  |  |  |
| **Men** | **Study-based mobility** | **NO** | **YES** |  | **Study-based mobility** |  |
|  | **NO** | ******* | ******* |  | NO vs YES [Women] | NS |
|  | **YES** | ******* | ******* |  | NO vs YES [Men] | * |

| **b)** | | **Women** | |  |  | |
| --- | --- | --- | --- | --- | --- | --- |
|  |  | **Work-based mobility** | |  |  |  |
| **Men** | **Work-based mobility** | **NO** | **YES** |  | **Work-based mobility** |  |
|  | **NO** | ******* | ****** |  | NO vs YES [Women] | * |
|  | **YES** | ******* | ******* |  | NO vs YES [Men] | NS |

Notes: a) study-related mobility; b) work-related mobility.
^***, **, *^ represent significance at the 0.01, 0.05 and 0.1 levels.

Table 10 Interactive variable coefficient comparison tests (t-tests) – Gender vs Collaboration

| **a)** | | **Women** | |  |  | |
| --- | --- | --- | --- | --- | --- | --- |
|  |  | **Collaboration with own inst.** | |  |  |  |
| **Men** | **Collaboration with own inst.** | **NO** | **YES** |  | **Collaboration with own inst.** |  |
|  | **NO** | *** | NS |  | NO vs YES [Women] | ******* |
|  | **YES** | *** | *** |  | NO vs YES [Men] | ******* |

| **b)** | | **Women** | |  |  | |
| --- | --- | --- | --- | --- | --- | --- |
|  |  | **Collaboration with own country** | |  |  |  |
| **Men** | **Collaboration with own country** | **NO** | **YES** |  | **Collaboration with own country** |  |
|  | **NO** | *** | NS |  | NO vs YES [Women] | ******* |
|  | **YES** | *** | *** |  | NO vs YES [Men] | ******* |

| **c)** | | **Women** | |  |  | |
| --- | --- | --- | --- | --- | --- | --- |
|  |  | **Collaboration within Africa** | |  |  |  |
| **Men** | **Collaboration within Africa** | **NO** | **YES** |  | **Collaboration within Africa** |  |
|  | **NO** | *** | NS |  | NO vs YES [Women] | ******* |
|  | **YES** | *** | NS |  | NO vs YES [Men] | ****** |

| **d)** | | **Women** | |  |  | |
| --- | --- | --- | --- | --- | --- | --- |
|  |  | **Collaboration outside Africa** | |  |  |  |
| **Men** | **Collaboration outside Africa** | **NO** | **YES** |  | **Collaboration outside Africa** |  |
|  | **NO** | *** | NS |  | NO vs YES [Women] | ******* |
|  | **YES** | *** | *** |  | NO vs YES [Men] | ******* |

Notes: a) with own inst.; b) with own country; c) within Africa; d) outside Africa.
^***, **, *^ represent significance at the 0.01, 0.05 and 0.1 levels.
